# Is there need for routine CT colonography after CT-verified uncomplicated diverticulitis of the sigmoid colon?

**DOI:** 10.1016/j.ejro.2021.100341

**Published:** 2021-03-31

**Authors:** Tormund Njølstad, Victoria Solveig Young, Anders Drolsum, Johann Baptist Dormagen, Bjørn Hofstad, Anselm Schulz

**Affiliations:** a Department of Radiology and Nuclear Medicine, Oslo University Hospital Ullevål, Oslo, Norway; b Department of Radiology, Haukeland University Hospital, Bergen, Norway; c Department of Gastroenterology, Oslo University Hospital, Oslo, Norway

**Keywords:** Diverticulitis, CT colonography, Colorectal cancer

## Abstract

**Background:**

Following an episode of acute diverticulitis, surgical guidelines commonly advise routine colonic follow-up to rule out underlying malignancy. However, as a CT of the abdomen is frequently performed during clinical work-up, the routine need for colonic follow-up has become debated.

**Purpose:**

To evaluate the need for routine CT colonography after an episode of CT-verified uncomplicated sigmoid diverticulitis to rule out underlying colorectal malignancy.

**Material and methods:**

This study retrospectively evaluated 312 patients routinely referred to colonic evaluation by CT colonography following an episode of acute diverticulitis. Patients were excluded if lacking diagnostic CT of the abdomen at time of diagnosis, if presenting with atypical colonic involvement, or if CT findings were suggestive of complicated disease (e.g., abscess or perforation). CT colonography exams were routinely reviewed by experienced abdominal radiology consultants on the day of the procedure. If significant polyps were detected, or if colorectal malignancy could not be excluded, patients were referred to same-day optical colonoscopy. For these patients, medical records were reviewed for optical colonoscopy results and histology reports if applicable.

**Results:**

Among 223 patients with CT-verified uncomplicated sigmoid diverticulitis, no patients were found to have underlying colorectal malignancy. 27 patients were referred to optical colonoscopy based on CT colonography findings. 18 patients consequently underwent polypectomy, all with either hyperplastic or adenomatous histology.

**Conclusions:**

This study indicates that routine colonic evaluation by CT colonography following an episode of CT-verified uncomplicated sigmoid diverticulitis may be unwarranted, and should arguably be reserved for patients with protracted or atypical clinical course.

## Highlights

• Patients with diverticulitis are commonly referred to colonic follow-up.
• None with CT-verified uncomplicated sigmoid diverticulitis had underlying cancer.
• Routine colonic follow-up should be reserved for select patients.

## 1 Introduction

Acute diverticulitis, an inflammation of a colonic diverticulum, is a common cause of abdominal pain in an in-hospital setting, and represents a significant burden on health care resources in developed countries [1]. Clinical course is generally uncomplicated. However, a subset of patients develop a more complicated disease as a result of abscess, fistula formation, bowel obstruction, peritonitis or perforation [2]. Thus, CT of the abdomen has emerged as the diagnostic test of choice to verify the diagnosis of acute diverticulitis and detect complications requiring prompt surgical intervention. For this, CT has high sensitivity, specificity, positive and negative predictive values (all reported well above 95 % in recent literature) [3].

Several studies have reported an increased risk of underlying colorectal cancer among patients presenting with acute diverticulitis [[4], [5], [6]]. On this basis, surgical guidelines commonly advise routine follow-up with colonoscopy after 4–8 weeks to rule out underlying malignancy [[7], [8], [9]]. However, such guidelines are arguably based on older studies without CT imaging being routinely applied in the diagnostic process, and where the clinical accuracy for diagnosing diverticulitis is reported as low [10,11]. Today, as a CT of the abdomen is commonly performed during clinical work-up to verify the diagnosis, the routine need for colonic follow-up has become debated. Several recent studies have found that the risk for underlying malignancy is low, and that routine colonic follow-up of uncomplicated diverticulitis should be reserved for selected patients (e.g., with protracted or atypical clinical course) [3,10,[12], [13], [14], [15]].

To our knowledge, no studies have evaluated the use of CT colonography as follow-up of CT-verified diverticulitis. As colonic follow-up by CT colonography represents our institutional routine, we set out to systematically review the results of these exams applied as follow-up for uncomplicated CT-verified diverticulitis.

## 2 Material and methods

For this study, we retrospectively reviewed all CT colonography exams performed on patients referred as follow-up for an episode of acute diverticulitis at our institution between 01.01.2012 and 31.12.2018. Our hospital is a university-affiliated specialist care center, catering to a large proportion of the population in Oslo, Norway, in both a secondary and tertiary care setting. Patients were identified by a protocol-specific search in our Radiology Information System (syngo Workflow, Siemens Healthcare, Erlangen, Germany). Our study was approved by the institutional review board, and by the Norwegian Data Protection Authority, and the need for individual patient consent was waived in this institutional quality-control setting.

### 2.1 Patient inclusion

All CT colonography exams were reviewed for their indication, and all patients not referred as follow-up for an episode of acute diverticulitis were excluded. All exams were then cross-referenced with our Radiology Information System to exclude patients lacking a diagnostic CT prior to referral. The diagnostic CT was reviewed to identify which colonic segment was involved, and to identify signs of complicated disease. Images were routinely evaluated using the Picture Archiving and Communication System applied at our radiology department (syngo Studio, Siemens Healthcare, Erlangen, Germany). For this study, uncomplicated diverticulitis was defined as CT findings of acute diverticular disease (e.g., diverticular disease, colonic wall thickening and signs of pericolic fatty stranding) without signs of localized or distant perforation, or abscess formation. Only patients with diverticulitis involving the sigmoid colon (and “transition zone” between the sigmoid colon and the distal descending colon or rectosigmoid colon) were included.

### 2.2 Imaging protocol

All follow-up CT colonography exams were obtained using a standardized protocol. Patient preparation routinely included two oral contrast tagging agents and a cleansing agent (the latter was omitted for patients over 75 years old). Tagging was performed using barium suspension (Tagitol V 40 %, 2 × 20 ml, Bracco, Milan, Italy) and diatrizoate (Gastrografin, 50 ml, Bayer, Milan, Italy), and cleansing using sodium picosulfate and magnesium citrate (CitraFleet, Casen Recordati, Utebo, Spain). Patients were given an intravenous antispasmodic, butylscopolamine (Buscopan, 20 mg/ml, 1 ml, Sanofi-aventis, Paris, France), two minutes before the exam. Colonic insufflation was achieved using a 6 mm rectal tube and an automated carbon dioxide insufflator (PROTOCO_2_L, E-Z-EM, Bracco, Milan, Italy) with target inflation pressure of 25 mmHg (reduced to 20 mmHg if the patient experienced discomfort or abdominal pain). Scans were obtained using initially a 64-slice CT scanner (Philips Brilliance, Philips Healthcare, Amsterdam, The Netherlands), and later a 128-slice CT scanner (Siemens SOMATOM Drive, Siemens Healthcare, Erlangen, Germany), with patients in both supine and prone position. Consistent image acquisition specifications were applied, using 40 mm collimation, with a slice thickness of 1 mm and an interval of 0.8 mm, 210 effective mAs as reference, 120 kV peak voltage, and field of view to fit the patient. For the prone position, a low-dose protocol was applied with 80 effective mAs as reference. For patients with difficulties performing the examination in the prone or supine position, scans in both laterals were obtained. Images were acquired using standard reconstruction algorithms, with multiplanar reconstruction with 3 mm slice thickness, as well as axial reconstructions with 0.625 mm slice thickness. Exams were reviewed using a Clinical Software Application tool at a dedicated workstation ensuring a fully automated virtual three-dimensional colonoscopy evaluation (IntelliSpace Portal, Philips Healthcare, Amsterdam, The Netherlands).

All reporting radiologists were experienced consultants with subspecialist training in abdominal radiology and with dedicated CT colonography training, and exams were routinely reviewed on the day of the procedure. In case of positive findings (e.g., findings suggestive of colorectal cancer, significant polyps or equivocal colonic findings), the patient was referred for same-day optical colonoscopy for further evaluation, treatment and/or treatment planning, as part of a collaboration between the department of radiology and department of gastroenterology at our institution. Polyps were routinely characterized in the CT colonography report according to size (≥10 mm as large, 6−9 mm as small, and ≤5 mm as diminutive) and morphology (sessile, pedunculated or flat), in addition to affected colonic segment and distance from the anal margin.

### 2.3 Evaluation

For this particular study, CT colonography reports were reviewed and categorized according to the classification system adapted by Zalis et al. [16]. Specifically, reports were categorized as either (1) normal colon or benign findings, with no polyps ≥6 mm size, (2) intermediate polyp (6−9 mm, <3 in number), (3) polyp possibly being advanced adenoma (≥10 mm size or ≥3 polyps, each 6−9 mm), or (4) colonic mass, likely malignant. The latter included cases where the reporting radiologist noted that colorectal cancer otherwise could not be excluded. Extracolonic findings were recorded, and only findings not present or reported on the diagnostic abdominal CT performed during clinical workup was considered relevant for this study. For patients with positive CT colonography findings, medical records were reviewed for same-day optical colonoscopy results, histological results (if applicable), and records of being diagnosed with and/or treated for colorectal cancer.

Study data were recorded and processed using Microsoft Office Excel 2010® (Microsoft Corporation, Redmond, WA USA) and evaluated using IBM SPSS Statistics® version 25.0 (IBM Corporation, Armonk, NY USA). The ‘rule of three’ was applied to estimate the upper 95 % confidence interval limit when no events were observed [17]. Expected number of colorectal cancers for our patient cohort was calculated by matching our patient cohort with age-specific incidences obtained from the Cancer Registry of Norway [18].

## 3 Results

**Fig. 1:**
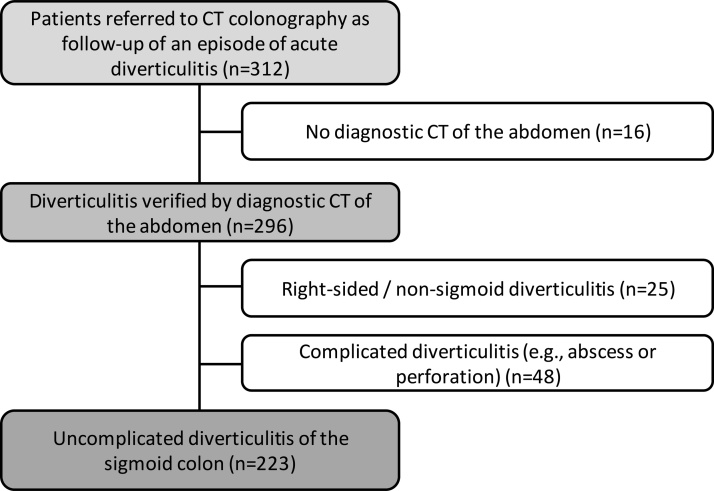
Flow of patients through our study from n = 312 patients referred for CT colonography following an episode of acute diverticulitis to n = 223 patients with uncomplicated CT-verified sigmoid diverticulitis included for further analysis.

**Fig. 2:**
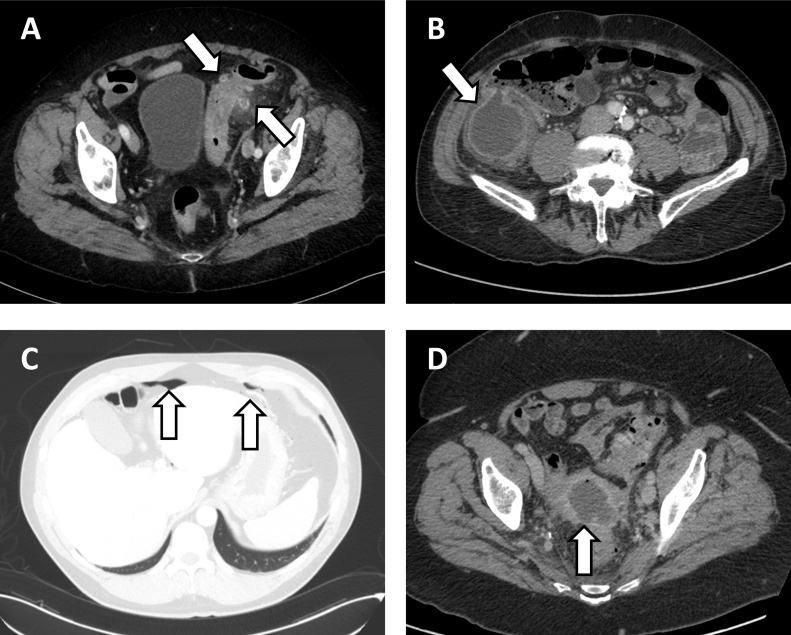
A selection of axial CT images of the abdomen. A) Example of uncomplicated sigmoid diverticulitis. Contrast-Enhanced Computed Tomography (CECT) shows sigmoid colon wall thickening, multiple diverticula and surrounding fatty stranding, suggestive of uncomplicated sigmoid diverticulitis. B) Example of right-sided diverticulitis. CECT shows a large diverticulum originating from the coecum, with surrounding fatty stranding, suggestive of an inflamed diverticulum. C) Example of perforated diverticulitis. CECT shows intraabdominal free air anteriorly beneath the diaphragm, suggestive of perforated hollow viscus. D) Example of complicated diverticulitis with abscess formation. CECT shows a 7 cm large fluid accumulation with a small air-fluid level, peripheral contrast enhancement and surrounding fatty stranding in relation to an inflamed sigmoid colon with multiple diverticula, suggestive of diverticulitis complicated with abscess formation.

Fig. 1 illustrates the patient flow in our study. A total of 312 patients were referred to a CT colonography exam as follow-up for an episode of acute diverticulitis at our institution between 01.01.2012 and 31.12.2018. 16 patients were excluded due to lack of diagnostic CT of the abdomen prior to referral (these patients were predominantly referred from primary care with a clinical suspicion of diverticulitis) and 25 patients were excluded due to atypical colonic involvement (see Table 1 for details). Furthermore, 48 patients were excluded due to radiological signs of complicated disease (i.e., abscess formation or perforation). This left 223 patients for further analysis. A selection of example CT images is presented in Fig. 2. Noteworthy, no specific imaging criteria based on e.g., asymmetric wall thickening, presence of lymphadenopathy, or length of sigmoid colonic segment involved was applied to select or discard patients from final analyses.

**Table 1:** Location of acute diverticulitis on diagnostic CT of the abdomen.

Location	Uncomplicated disease	Complicated disease	All patients
Coecum	1 (0.3 %)	0 (0.0 %)	1 (0.3 %)
Ascending colon	5 (1.7 %)	0 (0.0 %)	5 (1.7 %)
Right colonic flexure	1 (0.4 %)	0 (0.0 %)	1 (0.3 %)
Transverse colon	6 (2.0 %)	0 (0.0 %)	6 (2.0 %)
Left colonic flexure	2 (0.7 %)	1 (0.3 %)	3 (1.0 %)
Descending colon	6 (2.0 %)	3 (1.0 %)	9 (3.0 %)
Sigmoid colon (including transition zone to distal descending colon and rectum)	223 (91.4 %) a	48 (92.3 %)	271 (91.6 %)
Total	244 (82.4 %) a	52 (17.6 %)	296 (100 %)

a Only patients with uncomplicated diverticulitis affecting the sigmoid colon were included for further analysis (n = 223).

**Fig. 3:**
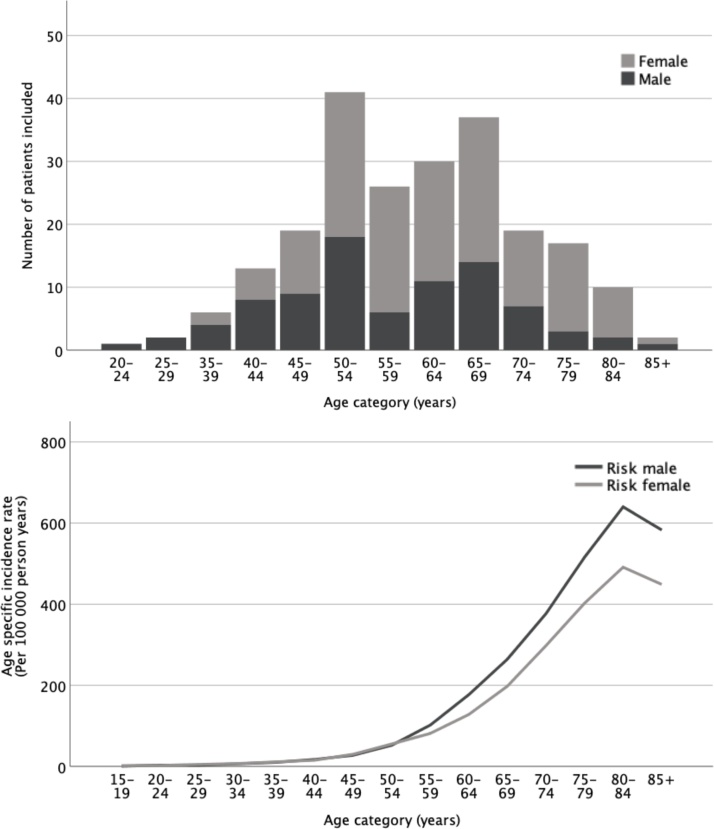
Age distribution for n = 137 female and n = 86 male patients with CT-verified uncomplicated sigmoid diverticulitis grouped by 5-year periods (bar chart), and corresponding age-specific incidence rates for colorectal cancer obtained from the Cancer Registry of Norway (line chart) [18].

Out of 223 included patients, 61.4 % were female and 38.6 % were male, with a collective mean age of 60.6 ± 12.3 years. The age distribution of included patients is presented in Fig. 3, along with corresponding age-specific incidence rates for colorectal cancer obtained from the Cancer Registry of Norway [18]. CT colonography was performed on average 65.5 days after the initial diagnostic CT (range 15–362 days). Volume computed tomography dose index (CTDIvol) for the full examination (including full- and low-dose scans) was estimated to 17.1 ± 6.2 mGy (data missing for 16 patients).

CT colonography exams were reviewed by 12 different abdominal radiologists, and 83 % of the exams were double-read. An overview of CT colonography findings is presented in Table 2. The exam was reported as negative for 196 patients, with no findings suggestive of underlying malignant colorectal disease and no significant polyps. For three patients, a follow-up CT colonography was recommended by the reporting radiologist, as the patients had substantial residual changes of diverticulitis. These follow-up exams were all negative. The remaining 27 patients had findings requiring additional investigation, and were routinely referred to same-day optical colonoscopy. Twenty patients were referred based on findings of polyps larger than 6 mm. Subsequent polypectomy was performed in 18 patients, and was omitted for two patients - one due to technical reasons and one due to severe comorbid cardiovascular disease. All resected polyps were either hyperplastic or adenomatous on histological evaluation. Nine patients were referred to optical colonoscopy based on colonic findings where underlying malignancy could not be excluded (two patients with small polyps also had colonic wall thickening where underlying malignancy could not be excluded). Among these patients, endoscopy revealed no malignant findings. Noticeably, when reviewing the 48 patients with complicated disease excluded from our final analyses, there were no malignant findings among this cohort.

**Table 2:** CT colonography findings among patients with CT-verified uncomplicated sigmoid diverticulitis, categorization adapted to classification system by Zalis et al [16].

Category	CT colonography findings	Number of patients (percent of total)
1	No malignant findings or significant polyps (≥6 mm size)	196 (87.9 %)
2	Intermediate polyp (6−9 mm, <3 in number)	10 (4.5 %)*
3	Polyp possibly being advanced adenoma (≥10 mm size or ≥3 polyps, each 6−9 mm)	10 (4.5 %)
4	Colonic mass, likely malignant, or where colorectal cancer otherwise could not be excluded	9 (4.0 %)*
	Total number of patients included	223 (100 %)

* Two of the patients with intermediate polyps also had colonic wall thickening where underlying malignancy could not be excluded.

Three patients had relevant extracolonic findings. For one patient, the CT colonography exam also covered the mediastinum, revealing enlarged lymph nodes, that had not been scanned by the diagnostic CT. The patient was diagnosed with sarcoidosis during subsequent clinical work-up. For a second patient, the report included possible signs of nephritis, and a third small amounts of pleural fluid. The last two findings had no clinical implications.

In total, out of all 223 included CT colonography exams referred as follow-up for an episode of CT-verified uncomplicated sigmoid diverticulitis, no patients were found to have underlying colorectal malignancy. The 95 % confidence interval for risk of underlying malignancy was estimated to 0-0.013. For comparison, the adjusted age-specific incidence of colorectal cancer among our cohort of 223 patients was estimated to 0.4 cases per year.

## 4 Discussion

Routine colonic evaluation after an episode of diverticulitis has been standard of care for several decades. However, recommendations are largely based on studies conducted before the wide-spread use of cross-sectional imaging, and at a time when diverticulitis primarily was diagnosed based on clinical findings and contrast enema studies [19]. However, the clinical diagnostic accuracy was considered low, exemplified by up to 37 % of diagnoses being changed with the application of cross-sectional imaging [20]. With considerable improvements in imaging technology over the last decades, and now routine use of high-resolution multi-detector scanning, CT imaging has a sensitivity and specificity reported well above 95 % for the diagnosis of diverticulitis, and for detecting complications, [3]. This raises the question of whether routine colonic follow-up is really necessary.

Our study demonstrates that among 223 patients referred to a follow-up examination by CT colonography following an episode of CT-verified uncomplicated sigmoid diverticulitis, there were no underlying malignancies detected. On this basis, we postulate that the risk of underlying malignancy is low, and routine use of colonic follow-up may be unwarranted in uncomplicated cases when the diagnosis has been verified by cross-sectional imaging (CT). Our findings are in line with several recent studies [3,10,[12], [13], [14], [15]]. Noticeably, a recent systematic review and meta-analysis by Sharma et al. [10], found that among 1497 patients with uncomplicated diverticulitis, cancer was found in only five (proportional estimate of risk 0.7 %). Among 79 patients with complicated disease, however, cancer was found in six (proportion estimate of risk 10.8 %). Thus, the authors conclude that the risk of malignancy after a radiologically proven episode of acute uncomplicated diverticulitis is low, and that in the absence of other indications, routine colonoscopy may not be necessary. Patients with complicated diverticulitis may still have a significant risk of colorectal cancer, and subsequent colonic evaluation may be warranted.

Furthermore, when comparing the age distribution of our patient cohort referred to colonic follow-up by CT colonography to the age-specific incidence for colorectal cancer, we find that many patients are referred at an age where the baseline risk for colorectal malignancy is low (Fig. 3). Thus, assuming that a slightly increased risk for colorectal malignancy is present, the overall risk for malignancy would arguably still be low. Moreover, this may be an indication that we need better tools for risk stratification and patient selection to select the right patients for colonic follow-up.

In general, colonic follow-up is performed by optical colonoscopy. However, we predominantly evaluate these patients by CT colonography due to higher patient acceptability and technical feasibility within this patient group. With demonstrated safety [21], this is also in line with recommendations from the European Society of Gastrointestinal and Abdominal Radiology [22,23]. CT colonography has been shown to be accurate in a screening setting, and to compare favourably with optical colonoscopy in detecting clinically relevant lesions [24]. Furthermore, studies investigating primary optical colonoscopy and CT colonography screening strategies have shown similar detection rates for advanced neoplasia, although smaller number of polypectomies performed in the patient group investigated by CT colonography [25].

Some studies have shown an increased risk of colon cancer among patients with diverticular disease, attributed to shared etiological factors [4]. A population-based, case control study by Stefanson et al. investigating patients discharged with a diagnosis of sigmoid diverticulitis in Uppsala, Sweden, between 1965-83 found an increased risk of left-sided colon cancer, suggesting a causal relationship [5]. This was supported by a more recent Danish 18-year nationwide cohort study assessing 40 496 patients [6]. However, these studies are based on patients diagnosed with diverticulitis, and not necessarily verified by CT imaging. Due to the overlap in symptoms with colorectal cancer, this represents a potential selection bias for these studies [2]. Furthermore, in the latter study, 34 % of the included patients diagnosed with colon cancer received this diagnosis prior to their first diagnosis of diverticulitis.

Patients with diverticulitis affecting the non-sigmoid colon are substantially less prevalent in the Norwegian population. These patients were excluded from our study, as we believe that colonic follow-up may be warranted for these patients due to the low prevalence of atypical diverticulitis and thus potentially higher risk for malignancy. Additional studies are, however, needed to precisely determine this underlying risk.

In our study, 9 % of the patients with uncomplicated diverticulitis (20 of 223 patients) were found to have significant polyps, and 18 underwent subsequent polypectomy. As reviewed by Pickhardt et al., similar rates of polyp detection has been shown in a screening setting, with a reported 13–15 % prevalence for polyps of all sizes and 5–7 % prevalence for large polyps (≥10 mm) [11]. Some studies have, however, shown an increased prevalence of polyps in patients with diverticular disease [10,26]. And, as colonoscopic polypectomy has shown to lead to a lower-than-expected incidence of colorectal cancer [27], one might argue that patients with diverticular disease should be referred for colonic evaluation. On the other hand, as diverticulosis of the colon is a very common condition reported to affect up to 50 % of patients older than 60 years of age [28], one might argue that colonic evaluation should rather be conducted according to an established population screening program.

Our study is not without limitations. With our retrospective approach, only patients who were referred to (and underwent) a follow-up CT colonography were included, with the possibility of introducing a selection bias. However, as this represents an institutional routine, we assume that only an insignificant number of patients have been missed. Ideally, a prospective population-based approach should be applied in this regard. Furthermore, as our recorded outcome (colorectal cancer) is rare, and as our study had no such observed events, this risk of malignancy among our patient cohort remains unknown. By comparison, the estimated age-adjusted incidence of colorectal cancer among our patient cohort based on numbers from the Cancer Registry of Norway was 0.4 cases per year. Furthermore, reports from a screening setting involving 8848 individuals found 33 colorectal cancers by flexible sigmoidoscopy (i.e., 1 per 268 exams) [29]. Thus, a large sample size would be required to more precisely estimate the underlying risk of colorectal malignancy given an episode of diverticulitis. Despite this, our study indicates that the short-term risk of underlying colorectal malignancy is low, given that diagnostic CT imaging has been applied. Noticeably, our study does not address the long-term risk for colorectal malignancy given an episode of CT-verified uncomplicated diverticulitis. Larger studies, ideally with a prospective population-based approach, are needed for a more precise estimate of the underlying risk of malignancy and the underlying risk factors for malignancy among patients presenting with acute diverticulitis.

In conclusion, this study shows that among 223 patients diagnosed with CT-verified uncomplicated diverticulitis of the sigmoid colon, no cancers were detected by CT colonography during routine follow-up. Thus, our study indicates that routine colonic evaluation by CT colonography following an episode of CT-verified uncomplicated sigmoid diverticulitis may be unwarranted, and should arguably be reserved for patients with protracted or atypical clinical course.

## Ethical statement

This study was approved by the institutional review board, and by the Norwegian Data Protection Authority, and the need for individual patient consent was waived in this institutional quality-control setting.

## Funding sources

This research did not receive any specific grant from funding agencies in the public, commercial, or not-for-profit sectors.

## CRediT authorship contribution statement

**Tormund Njølstad:** Data curation, Investigation, Writing - original draft. **Victoria Solveig Young:** Conceptualization, Data curation, Investigation, Writing - review & editing. **Anders Drolsum:** Validation, Resources, Writing - review & editing. **Johann Baptist Dormagen:** Supervision, Writing - review & editing. **Bjørn Hofstad:** Conceptualization, Validation, Writing - review & editing. **Anselm Schulz:** Conceptualization, Writing - review & editing, Supervision, Project administration.

## Declaration of Competing Interest

The authors report no declarations of interest
